# A novel fibrinogen variant in a Chinese pedigree with congenital dysfibrinogenemia caused by FGA P. Arg38Thr mutation: A case report

**DOI:** 10.1097/MD.0000000000012697

**Published:** 2018-10-05

**Authors:** Ruimin Cai, Yi Li, Wenyang Wang, Xue Gao, Meirong Liu, Youxiang Diao, Yi Tang, Qiang Feng

**Affiliations:** a Department of Clinical Laboratory, Central Hospital of Taian; b Taishan Medical University; c Shandong Agricultural University, Taian, China.

**Keywords:** dysfibrinogenemia, fibrinogen, missense mutation

## Abstract

**Rationale:**

Congenital dysfibrinogenemia (CD) is characterized by altered functional properties of the fibrinogen; people who suffer from CD often have a low activity of fibrinogen and the mutation in the genomic DNA.

**Patient concerns:**

A 6-year-old child was examined with a low activity of fibrinogen measured by Von Clauss method and PT-derived method which indicated a normal level of fibrinogen; this abnormality was also detected in her mother. The genomic DNA of all the family members was extracted, and all exons of 3 fibrinogen genes which encode fibrinogen alpha chain (FGA), fibrinogen beta chain (FGB), and fibrinogen gamma chain (FGG) were amplified by polymerase chain reaction (PCR), in addition, sanger sequencing, homologous sequence alignment and bioinformatics software were performed for the further analysis.

**Diagnoses:**

CD in this pedigree is associated with c.113G>C in the exon 2 of FGA which caused Arg38Thr mutation.

**Outcomes:**

The child and her mother showed a low plasma concentration of fibrinogen measured by Von Clauss method, whereas a normal result measured by PT-derived method; finally, c.113G>C in the exon 2 of FGA was detected in the pedigree which caused Arg38Thr mutation and it is the first report on a pedigree with CD caused by AαArg38Thr.

**Lessons:**

This case gives us the lesson that not all patients with CD showed typical symptoms and laboratory test results; the result of fibrinogen concentration and antigen which is tested by Von Clauss method and immunoturbidimetric assay is various according to the condition of each CD patient.

## 1 Introduction

Fibrinogen disorder is a rare disease that may affect the quantity of fibrinogen or the altered functional properties of the protein, even both of them. Congenital dysfibrinogenemia (CD) is characterized by fibrinogen dysfunction caused by an abnormality in fibrinogen molecular structure,^[1]^ which has a normal antigen level but a low function activity of fibrinogen. Based on the inconsistence between the antigen levels and fibrinogen activity, CD can be preliminarily diagnosed.^[2]^ The majority of CD patients is inherited as a result of an autosomal-dominant point mutation^[3]^; it can also be codominant or autosomal recessive inheritance. Fibrinogen is the most abundant coagulation factor in plasma, and soluble fibrinogen is converted into insoluble fibrin with thrombin. Fibrinogen is not only involved in the blood coagulation, but also plays a major part in platelet aggregation and adhesion with binding to glycoprotein IIb/IIIa receptor on platelets.^[4]^ Fibrinogen is a plasma glycoprotein composed of 2964 amino acids, forming a symmetrical hexameric structure (Aα, Bβ, γ)_2_,^[5]^ which is encoded by 3 fibrinogen genes: FGA, FGB, and FGG; thus, the heterozygous missense mutations existing in most patients occur in 1 of the 3 genes,^[6]^ and these mutation sites are mainly located on the exon 2 of FGA and exon 8 of FGG. Research suggests CD is responsible for thromboembolic phenomena, bleeding, or both of them, while more than a few patients are asymptomatic,^[7]^ but the risk of bleeding or thrombosis is higher than that of normal people; moreover, CD patients may suffer from obstetric complications, such as placental abruption, spontaneous abortion, and so on. In this paper, the phenotypic and genotypic analysis of a family with CD was conducted to preliminarily explore the pathogenesis of CD in the family that we discovered.

## 2 Case presentation

**Figure 1:**
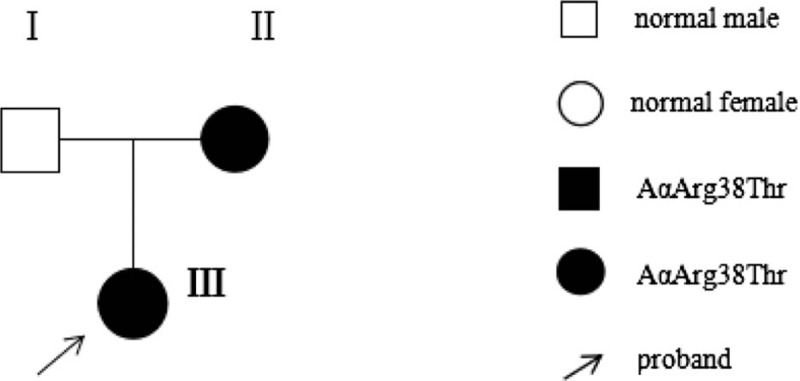
Pedigree of the family.

The proband (III) was a 6-year-old child from Shandong Province, visited for symptoms of cough and fever, who had no history of bleeding tendency or thrombosis, routine coagulation tests showed a low plasma concentration of fibrinogen measured by Von Clauss method, and a normal result measured by PT-derived method. She had normal activated partial thromboplastin time (APTT), slightly prolonged prothrombin time (PT), and extremely prolonged thrombin time (TT) on a Sysmex automatic blood coagulation analyzer (Sysmex CA7000, Japan). Her mother (II) had the same result, who had coagulation disorders during the pregnancy; the father of proband (I) had a normal coagulation test results; the pedigree of the family was shown in Figure 1, and the results of routine coagulation tests of the family members were shown in Table 1. Finally, the informed consent was obtained from all the family members for the coagulation tests, mutation analysis, and publication of this report.

**Table 1:**
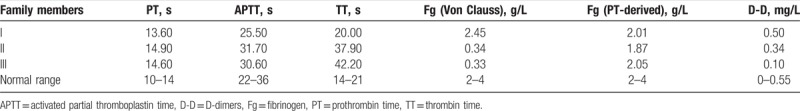
The results of routine coagulation tests of the family members.

## 3 Diagnostic methods

### 3.1 Polymerase chain reaction amplification

Genomic DNA was extracted from fresh blood samples using the DNA extraction Kit (TIANGEN Biotech Co. Ltd, Beijing, China). The concentration and purity of the genomic DNA extraction were measured on the DS-11 ultra-micronuclear protein assay apparatus (Denovix, USA). All exons and flanking sequence of the 3 fibrinogen genes (FGA, FGB, and FGG) were amplified by polymerase chain reaction (PCR) using specific primers; the sequences of all the primers were referred to the other study,^[8]^ and synthesized by TSINGKE Bioscience & Technology Company (Beijing, China). PCR was performed with a mixture reaction of 20 μL, which contained 10 μL of PCR Master Mix, 6 μL of ddH_2_O, and 1 μL of 20 μmol/L forward primer, 1 μL of 20 μmol/L reverse primer, 2 μL of DNA sample, using a PCR system where the thermal cycling profile is as follows: 95°C for 5 minutes, 95°C for 30 seconds, annealing at 55°C for 45 seconds, extension at 72°C for 45 seconds, followed by 32 cycles, and elongation at 72°C for 10 minutes on the DNA Thermal Cycler (A300 Thermal Cycler, LongGene, Hangzhou, China). The PCR products were identified on 1% agarose gel, and purified by Easy Pure Quick Gel Extraction Kit (TransGen Biotech Co. Ltd, Beijing, China) according to the manufacture's instructions.

### 3.2 DNA sequence analysis

The concentration of DNA extraction and the purity ratio of OD260/280 were measured on the DS-11 ultra-micronuclear protein assay apparatus. The concentration of the DNA sample was 100 ng/μL; the purity ratio range was 1.90 to 2.10; DNA sequencing was performed by TSINGKE Bioscience & Technology Company. The sequencing results and corresponding Genbank (FGA: NG-008832.1, FGB: NG-008833.1, and FGG: NG-008834.1) sequences were analyzed by Megalign software to identify mutations.

### 3.3 Bioinformatics software analysis

Functional analysis of the mutation site was predicted by PolyPhen-2 (http://genetics.bwh.harvard.edu/pph2/) and Mutation Taster (http://www.mutationtaster. Org/).

### 3.4 Homogeneous sequence alignment

Conservative analysis of the amino acid sequences of Aα chain between different species was performed by using the ClusterX software.

## 4 Results

### 4.1 DNA analysis

**Figure 2:**
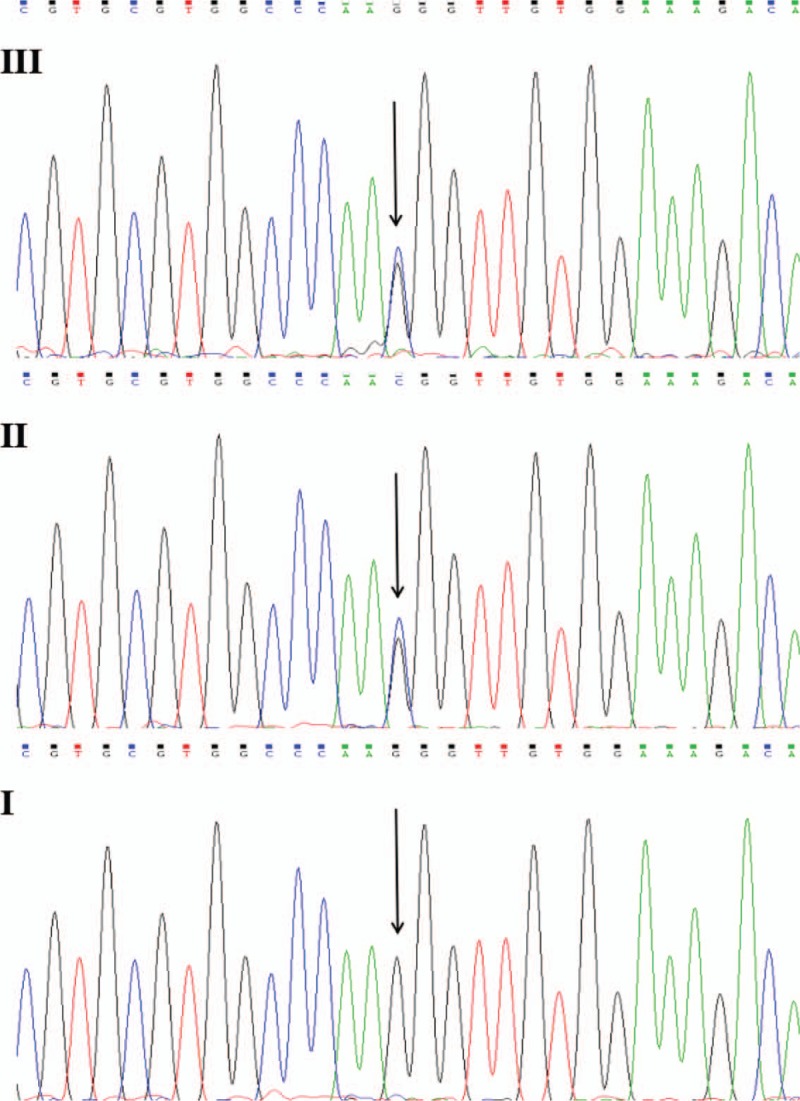
The sequences of exon 2 of FGA in the family members. A heterozygous mutation G → C in the exon 2 of FGA at nucleotide position 113 (cDNA numbered) was found in the proband and her mother; no mutation was revealed in the father of the proband (III), proband (II) mother of the proband; (I) father of the proband.

The nucleotides of fibrinogen FGA, FGB, and FGG genes were analyzed by sanger sequencing; a heterozygous G → C replacement in the exon 2 of FGA at nucleotide position 113 (cDNA numbered) was found in the proband, resulting in a missense mutation of Arg38Thr of the Aα chain. Her mother had the same mutation site. There was no other mutation found in the exons of the FGA, FGB and FGG, and no mutation was revealed in the father of the proband (Fig. 2).

### 4.2 Bioinformatics software analysis

**Figure 3:**
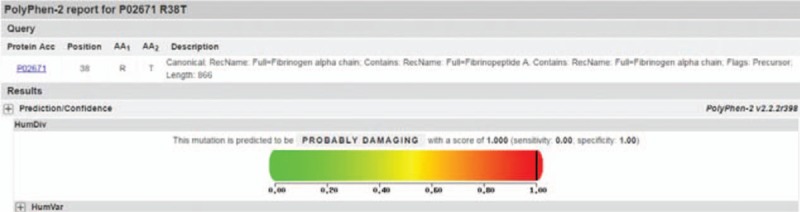
Bioinformatics analysis of FGA Arg38Thr by PolyPhen-2.

Based on the mutation Arg38Thr in the exon 2 of FGA, PolyPhen-2 scored 1 point, which indicates that this mutation is probably damaging (Fig. 3); the predicted result by Mutation Taster is disease-causing.

### 4.3 Homogeneous sequence alignment

**Figure 4:**
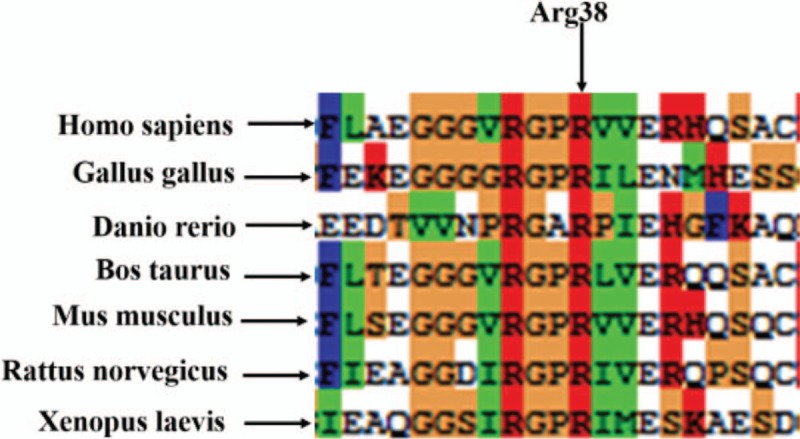
Homogeneous sequence alignment of Arg38 in the exon 2 of FGA among species. Homo sapiens, NCBI Reference Sequence: NP_000499.1; Gallus gallus, NCBI Reference Sequence: NP_990687.2; Danio rerio, NCBI Reference Sequence: NP_001181918.1; Bos taurus, NCBI Reference Sequence: NP_001028798.1; Mus musculus, NCBI Reference Sequence: NP_034326. 1; Rattus norvegicus, NCBI Reference Sequence: NP_434684.1; Xenopus laevis, NCBI Reference Sequence: NP_001080329.1.

The mutation site in the variant of Arg38Thr in the exon 2 of FGA is highly conservative among species (Fig. 4).

## 5 Discussion

Fibrinogen, platelets, coagulation factors, and enzymes are involved in the complicated interactions of the blood coagulation. The interactions include the initiation of thrombin, the propagation of thrombin, fibrin formation, and stabilization from fibrinogen and fibrinolysis.^[9–10]^ Genetic defects of fibrinogen can cause abnormal release of peptides A and B from the α and β chains, disturbance of polymerization of the fibrin monomers, fibrin cross-linking disorders, and abnormality of fibrinolysis of the fibrin gel.^[4]^ As other review reported, the sequence 17 Gly-18 Pro-19 Arg of fibrin α-chain binds to the complementary polymerization pocket in the γ-chain, which is responsible for the formation of the fibrin gel network.^[11]^ In this paper, FGA P. Arg38Thr mutation cause delayed polymerization of Aα chains and alterations in binding to the D domain of another γ chain, but patients in this pedigree are asymptomatic. Different mutation sites may lead to different or same pathogenesis and clinical manifestations, for example, AαArg16Ser and Arg16Cys mutation may result in abnormal protofibril assembly and disrupt E:D domain interactions^[12]^; however, different patient would suffer from asymptomatic, bleeding, or thrombosis. In addition, AαGly17 Val mutation caused polymerization of the fibrin monomers disorders with delayed wound healing^[13]^; the mutation of AαGlu11Gly reduces fibrinogen's binding with thrombin.^[14]^ Aα328 is related to α-chain cross-linking, so α-polymer formation was distinctly impaired when AαGln328Pro is caused.^[15]^ Aα554Arg→Cys mutation and Aα532Ser→Cys mutation are associated with thrombotic manifestations; BβArg14Cys mutation should be responsible for inherited thrombophilia.^[16–18]^

Coagulation profile and clinical manifestations are used as the initial screen for patients with CD, but not all patients showed typical coagulation test results and symptoms; clotting test results of this pedigree are emblematical, but the proband of this pedigree complained of cough for 10 days, and was diagnosed with bronchial pneumonia and mycoplasma infection; her mother was asymptomatic. However, sometimes fibrinogen concentration tested by Von Clauss method and immunoturbidimetric assay are reduced or normal, and even raised; unfortunately, 2 of the results above can occur simultaneously; in some particular cases, it is easy to escape diagnosis or be misdiagnosed. As a result, gene sequencing improves the chances of diagnosis and treatment of CD.

According to the reports on CD, Aα38Arg is a mutation hotspot, whereas the mutation sites located on Bβ and γ-chains are concentrated on highly conserved domains. It is proved that these domains play an important role in synthesis of fibrinogen. Although the currently discovered mutation sites are mainly located on the extron, mutations occurring on the intron might also have certain chain reactions with the development of CD, well it needs further study to confirm this hypothesis. In conclusion, Arg38Thr is responsible for the CD in this pedigree.

## 6 Conclusion

In summary, by the DNA sequencing, homologous sequence alignment and bioinformatics software analysis, the heterozygous missense mutation of c.113G>C in the exon 2 of FGA (Arg38Thr), we identified in this study, probably underlies the CD in this pedigree, and Arg38Thr was reported for the first time.

## Acknowledgments

We thank all the family members in the pedigree of our study for their cooperation, and experts in Department of Clinical Laboratory, Central Hospital of Taian and Shandong Agricultural University.

## Author contributions

**Data curation:** Meirong Liu.

**Investigation:** Xue Gao.

**Methodology:** Yi Li.

**Resources:** Yi Tang.

**Software:** Wenyang Wang.

**Supervision:** Youxiang Diao, Yi Tang.

**Validation:** Qiang Feng.

**Writing – original draft:** Ruimin Cai.
